# Saxagliptin Cardiotoxicity in Chronic Heart Failure: The Role of DPP4 in the Regulation of Neuropeptide Tone

**DOI:** 10.3390/biomedicines10071573

**Published:** 2022-07-01

**Authors:** Imre Vörös, Zsófia Onódi, Viktória Éva Tóth, Tamás G. Gergely, Éva Sághy, Anikó Görbe, Ágnes Kemény, Przemyslaw Leszek, Zsuzsanna Helyes, Péter Ferdinandy, Zoltán V. Varga

**Affiliations:** 1 Cardiometabolic Research Group and MTA-SE System Pharmacology Research Group, Department of Pharmacology and Pharmacotherapy, Semmelweis University, 1085 Budapest, Hungary; voros.imre@med.semmelweis-univ.hu (I.V.); onodi.zsofia@med.semmelweis-univ.hu (Z.O.); toth.viktoria@med.semmelweis-univ.hu (V.É.T.); gergely.tamas@med.semmelweis-univ.hu (T.G.G.); saghy.eva@med.semmelweis-univ.hu (É.S.); gorbe.aniko@med.semmelweis-univ.hu (A.G.); peter.ferdinandy@pharmahungary.com (P.F.); 2 HCEMM-SU Cardiometabolic Immunology Research Group, Semmelweis University, 1085 Budapest, Hungary; 3 MTA-SE Momentum Cardio-Oncology and Cardioimmunology Research Group, Semmelweis University, 1085 Budapest, Hungary; 4 Pharmahungary Group, 6722 Szeged, Hungary; 5 Szentágothai János Research Centre, University of Pécs, 7624 Pécs, Hungary; kemeny.agnes@pte.hu (Á.K.); zsuzsanna.helyes@aok.pte.hu (Z.H.); 6 Department of Pharmacology and Pharmacotherapy, Medical School, University of Pécs, 7624 Pécs, Hungary; 7 Department of Medical Biology, University of Pécs, 7624 Pécs, Hungary; 8 Department of Heart Failure and Transplantology, Cardinal Stefan Wyszyński National Institute of Cardiology, 04-628 Warszawa, Poland; przemyslaw.leszek@ikard.pl; 9 PharmInVivo Ltd., 7629 Pécs, Hungary

**Keywords:** saxagliptin, neuropeptide Y, substance P, neuropeptides, diabetes, heart failure, cardiotoxicity, cardiomyopathy

## Abstract

Dipeptidyl-peptidase-4 (DPP4) inhibitors are novel medicines for diabetes. The SAVOR-TIMI-53 clinical trial revealed increased heart-failure-associated hospitalization in saxagliptin-treated patients. Although this side effect could limit therapeutic use, the mechanism of this potential cardiotoxicity is unclear. We aimed to establish a cellular platform to investigate DPP4 inhibition and the role of its neuropeptide substrates substance P (SP) and neuropeptide Y (NPY), and to determine the expression of DDP4 and its neuropeptide substrates in the human heart. Western blot, radio-, enzyme-linked immuno-, and RNA scope assays were performed to investigate the expression of DPP4 and its substrates in human hearts. Calcein-based viability measurements and scratch assays were used to test the potential toxicity of DPP4 inhibitors. Cardiac expression of DPP4 and NPY decreased in heart failure patients. In human hearts, DPP4 mRNA is detectable mainly in cardiomyocytes and endothelium. Treatment with DPP4 inhibitors alone/in combination with neuropeptides did not affect viability but in scratch assays neuropeptides decreased, while saxagliptin co-administration increased fibroblast migration in isolated neonatal rat cardiomyocyte-fibroblast co-culture. Decreased DPP4 activity takes part in the pathophysiology of end-stage heart failure. DPP4 compensates against the elevated sympathetic activity and altered neuropeptide tone. Its inhibition decreases this adaptive mechanism, thereby exacerbating myocardial damage.

## 1. Introduction

Heart failure is a complex cardiovascular disease with high mortality rate and increasing prevalence [1]. Decreased cardiac output, when the heart is unable to provide the required perfusion to the peripheral tissues, is a critical consequence of heart failure. This event induces various compensatory mechanisms in order to maintain sufficient perfusion of vital organs. Important aspects of these compensatory processes are increased activity of the sympathetic nervous system and the consequently increased release of the sympathetic transmitters and co-transmitters (e.g., neuropeptide Y (NPY) and substance P (SP)), besides activation of the renin-angiotensin-aldosterone system, endothelin release, and inflammatory mechanisms [1]. In the short term, these processes will maintain tissue perfusion; however, in the long term, abnormalities in cellular signaling lead to inflammation, fibrosis, and cell death, which strongly promote the remodeling process, thus creating a vicious cycle to trigger the progression of heart failure.

The neuropeptide SP (acting through the neurokinin 1 receptor) is mainly expressed in the heart by C-fiber sensory nerves, innervating coronary arteries, intrinsic nerve bundles, coronary endothelial cells, and cardiomyocytes [2,3,4,5,6,7]. SP has been shown to play a detrimental role in various cardiovascular events, e.g., adverse cardiac remodeling, inflammation, necrosis, and fibrosis [8,9,10,11,12]. NPY is the most abundant neuropeptide in the heart, which exerts its actions through five known receptors (Y1 to Y5) [13]. NPY is expressed in the intracardiac ganglia, sympathetic nerves projecting to blood vessels, intrinsic parasympathetic cardiac neurons, and cardiomyocytes [14]. NPY was identified as a prognostic indicator of mortality in myocardial infarct patients [15].

The management of heart failure is still a challenging task for healthcare professionals, especially when co-morbidities are present in patients, e.g., type 2 diabetes [16]. Dipeptidyl-peptidase IV (DPP4) inhibitors are in general well-tolerated and relatively new options in the pharmacotherapy of type 2 diabetes [17,18]. It was discovered in the 1990s that the inactivation of the DPP-4 enzyme raised the levels of the incretin hormones, which in turn lowered the circulating glucose concentration and improved glycemia in animals [19]. In the following two decades, various small molecule DPP4 inhibitor substances have been developed and tested in subsequent clinical studies for the therapy of type 2 diabetes [19] and for other cardiovascular comorbidities [20,21,22]. The regulatory authorities have approved five of the DPP-4 inhibitors so far. Currently, these drugs are used as second- or third-line medications in the treatment of type 2 diabetes after metformin and sulphonylureas. These substances facilitate insulin secretion through increasing the level of glucagon-like peptide-1 indirectly by inhibiting DPP4, an enzyme responsible for the degradation of incretins (glucose-dependent insulinotropic peptide and glucagon-like peptide-1 [17,23]). DPP4 is a transmembrane exopeptidase, which cleaves dipeptides from the N-terminal site of its targets [24,25]. The cleavage results in the inactivation of the substrates in most cases, but sometimes changes in receptor affinity of certain substrates can also happen [25]. DPP4 has various types of substrates, e.g., incretins (glucagon-like peptide-1, glucose-dependent insulinotropic polypeptide), chemokines (e.g., stromal cell-derived factor 1), and neuropeptides (NPY, SP, peptide YY, and pancreatic polypeptide) [24,25]. Interestingly, it was recently identified that decreased circulating DPP4 activity in patients is associated with severe COVID-19 disease and is a strong prognostic biomarker of mortality [26]. The large randomized placebo-controlled phase 4 clinical trial (SAVOR-TIMI 53) on the cardiovascular safety of the DPP4-inhibitor saxagliptin surprisingly revealed that the heart-failure-associated hospitalization rate increased in the saxagliptin-treated group compared to controls [27]. The possible dangerous cardiovascular side effects of DPP4 inhibitors are included in the latest ESC heart failure guideline, which suggests avoiding the administration of gliptins in diabetic patients with heart failure [28]. Although this potentially cardiotoxic side effect of saxagliptin could considerably limit therapeutic use, the detailed mechanism by which DPP4 inhibitors may damage the heart is still unclear [27]. Therefore, we aimed to set up a relevant cell culture platform to mechanistically investigate the effect of DPP4 inhibition and the role of potentially important neuropeptide substrates such as SP and NPY. Moreover, we aimed to determine the expression of DPP4 and its substrates in the human heart and cell culture samples both at protein and mRNA levels.

## 2. Materials and Methods

### 2.1. Cell Culture

Human cardiac myocyte AC16 cell line was obtained from Merck (SCC109). Cells were plated on 6-well or 24-well plates (Thermo Fisher Scientific, Waltham, MA, USA) and maintained in growth medium (Dulbecco’s Modified Eagle’s Medium and Ham’s F-12 Nutrient Mixture-DMEM/F12; Thermo Fisher Scientific, Waltham, MA, USA) supplemented with 10% fetal bovine serum (FBS; EuroClone, Pero MI, Italy) and antibiotic (100 U/mL penicillin and 100 µg/mL streptomycin; Thermo Fisher Scientific, Waltham, MA, USA) at 37 °C in a humidified atmosphere of 5% CO_2_. Cells were cultured until 80–90% confluence, then used in experiments. Cells were kept in FBS-free DMEM/F12 medium during the whole experimental protocol.

### 2.2. Viability Measurements

In order to test the effect of different DDP4 inhibitors (alogliptin, linagliptin, saxagliptin, or vildagliptin) on cell viability, AC16 cells were treated with 500 nM of each gliptin in separate series for 24 h. Control group received DMSO vehicle.

In a separate experiment, cells were treated with 500 nM saxagliptin in the presence of different doses (5, 20, 50, and 100 nM) of NPY or SP. To achieve sufficient inhibition of DPP4 enzyme, saxagliptin was administered 1 h prior to neuropeptide treatment. Cell-culture-grade water containing DMSO and pure cell-culture-grade water were used as vehicles for saxagliptin and neuropeptides, respectively.

Cell viability was assessed by calcein viability assay [29]. After rinsing the plates with Dulbecco’s phosphate-buffered saline (D-PBS), cells were incubated with calcein AM solution (1 μM) for 30 min at room temperature, protected from light. Fluorescence intensity was detected by Varioskan Lux multimode microplate reader (Thermo Fisher Scientific, Waltham, MA, USA) at room temperature, by using excitation wavelength: 490 nm and emission wavelength: 520 nm. Results are shown as RFU [30].

### 2.3. Co-Culture of Neonatal Rat Cardiac Fibroblasts and Myocytes

Primary neonatal rat hearts were isolated from 1–3-day-old Wistar rats (~3–4 animal/24-well plate) as previously described [31]. Briefly, rats were disinfected with 70% ethanol and sacrificed by cervical dislocation. Hearts were rapidly removed from an abdominal approach and placed in ice-cold PBS then washed with fresh volumes of ice-cold PBS three times under laminar hood. Ventricles were separated from atria in a sterile Petri dish containing ice-cold PBS. Afterwards, ventricles were minced properly with fine forceps and collected in 0.25% trypsin (5 mL/heart) containing Falcon tubes. Tissue fragments were digested by trypsin for 25 min in 37 °C water bath. The cell suspension was resuspended every 5 min by a 5 mL pipette. After digestion, the cell suspension was centrifuged for 15 min at 250 rcf at 4 °C and the supernatant was removed gently. Cell pellets were resuspended in 10 mL of Gibco Dulbecco’s Modified Eagle Medium (DMEM) growth medium supplemented with 20% FBS. Cells were counted manually by hemocytometer then gently resuspended, divided equally into fibronectin-coated wells. Cells were maintained at 37 °C in a humidified atmosphere of 5% CO_2_ and 95% air in a CO_2_ incubator until ~100% confluence was reached. The medium was changed to DMEM growth medium supplemented with 10% FBS the day after preparation. The medium was changed again 6–8 h after the previous one to DMEM growth medium supplemented with 1% FBS. Cells were scored after the medium change. DMEM medium supplemented with 1% FBS was refreshed every 2–3 days until reaching the desired confluence and was used in scratch assay experiments.

### 2.4. Scratch Assay

Scratch assays were performed as reported previously [31]. Briefly, cells were seeded on 24-well plates, and were pretreated with 500 nM saxagliptin or its solvent for 1 h. After pretreatment, the cell monolayer was scratched with a 200 µL pipette tip in a 30° angle. The medium was discarded to remove cell debris from wells, and cells were washed two times with Hank’s Balanced Salt Solution (HBSS, Corning Inc., Somerville, MA, USA). Then, the cells were treated with a combination of saxagliptin and NPY or SP for 24 h in the same manner as described above. Cells were incubated for 24 h with the treatment solution at 37 °C in a humidified atmosphere of 5% CO_2_ and 95% air in a CO_2_ incubator. Images were taken at the start of the treatment (0 h) and 24 h after the scratch. The wounding area (unoccupied surface by cells) of the images was measured by ImageJ software [32] and expressed as percentage of baseline value (0 h). Data were collected from four independent experiments.

### 2.5. Human Heart Tissue Collection

The experiments were designed and implemented according to the ethical standards of the Declaration of Helsinki (1975). Patients gave their written informed consent to be involved in the study. The protocol was approved by the Polish Local Ethics Committee of the National Institute of Cardiology in Warsaw with the identification code IK-NPIA-0021-14/1426/18. Human tissue samples were collected in the Department of Heart Failure and Transplantology, Cardinal Stefan Wyszyński National Institute of Cardiology, Warszawa, Poland, as previously described [33]. Human hearts obtained from organ donors that were excluded from transplantation for various reasons were used as control (CON) samples. The donors with any relevant cardiovascular history or abnormalities were excluded in the present study. Failing hearts were obtained from patients suffering from end-stage heart failure of ischemic cardiomyopathy (ICM) or dilated cardiomyopathy (DCM). Clinical parameters of the human tissue samples used in Western blot (Supplementary Table S1) and in radioimmunoassay/ELISA (Supplementary Table S2) experiments are summarized in the supplementary files. Interventricular septum samples were collected during heart explantation, avoiding the inclusion of non-cardiac tissues, e.g., scar, adipose tissue, endocardium, epicardium, or coronary vessels. The samples were rinsed immediately in saline solution, blotted dry, frozen in liquid nitrogen, and kept at −80 °C until processing for further molecular assays. Another series of left ventricle samples was fixed in neutral buffered formalin and embedded in paraffin for histological assays.

### 2.6. RNA Scope^®^ In Situ Hybridization

In situ hybridization of the DPP4 enzyme mRNA was performed on tissue slides of interventricular septum harvested from human control hearts using RNA Scope^®^ Multiplex Fluorescent Kit v2 according to the manufacturer’s instructions (Advanced Cell Diagnostics, Newark, CA, USA). Briefly, as reported previously [34], 4 μm formalin-fixed paraffin-embedded tissue sections were pretreated with heat, H_2_O_2_, and protease prior to hybridization with the following target oligo probes: 3plex-Hs-Positive Control Probe (catalog number: 320861), 3plex-Negative Control Probe (catalog number: 320871), Hs-DPP4 (catalog number: 477541, accession no.: NM_001935.3), Hs-VIM-C2 (catalog number: 310441-C2, accession no.: NM_003380.3), Hs-CD68-C2 (catalog number: 560591-C2, accession no.: NM_001040059.1), Hs-PECAM1-O1-C3 (catalog number: 487381-C3, accession no.: NM_000442.4), and Hs-RYR2-C2 (catalog number: 415831-C2, accession no.: NM_001035.2). Cell-type-specific markers were used to identify cardiomyocytes with a probe recognizing the mRNA of ryanodine receptor 2 (RYR2) [35], endothelial cells with a probe recognizing the mRNA of platelet endothelial cell adhesion molecule 1 (PECAM-1) [36], fibroblast cells with a probe recognizing the mRNA of vimentin (VIM) [37,38], and macrophages with a probe recognizing the mRNA of cluster of differentiation 68 (CD68) [39], respectively. Next, preamplifier, amplifier, and HRP-labeled oligo probes were then hybridized sequentially, followed by signal development with TSA fluorophores (TSA-Cy3, TSA-FITC, Akoya Biosciences, Marlborough, MA, USA). Each sample was quality-controlled for RNA integrity with a positive control probe specific to the housekeeping genes and with a negative control probe. The specific RNA staining signal was identified as red/green dots. Nuclei were stained with 4′,6-diamidino-2-phenylindole (DAPI). Imaging was performed with a Leica DMI8 Confocal microscope (Leica, Wetzlar, Germany).

### 2.7. DPP4 Protein Expression in the Heart

In order to investigate whether DPP4 expression was altered at the protein level in the human heart samples, Western blot was performed. Frozen tissue samples from the interventricular septum were homogenized with a TissueLyser (Hilden, Germany) in 1× radio immunoprecipitation assay buffer (RIPA; Cell Signaling Technology, Danvers, MA, USA) supplemented with 1× HALT Protease and Phosphatase Inhibitor cocktail (Thermo Scientific, Waltham, MA, USA). Protein concentration of the samples was determined by bicinchoninic acid assay kit (Thermo Scientific, Waltham, MA, USA). Equal amounts of protein (25 µg) from each sample were mixed with 1/4 volume of Laemmli buffer containing β-mercaptoethanol (Thermo Scientific, Waltham, MA, USA) and were loaded on 4–20% Tris-glycine sodium dodecyl sulfate-polyacrylamide gels (Bio-Rad, Hercules, CA, USA). After separation by electrophoresis, proteins were transferred onto polyvinylidene difluoride membrane (Bio-Rad, Hercules, CA, USA) with Trans-Blot^®^ Turbo^TM^ Transfer System (Bio-Rad, Hercules, CA, USA). Membranes were blocked in 5% bovine serum albumin (Bio-Rad, Hercules, CA, USA) in Tris-buffered saline containing 0.05% Tween-20 (0.05% TBS-T; Sigma, St. Louis, MO, USA) for 2 h at room temperature. Afterwards, membranes were incubated with anti-DPP-4 primary antibody overnight at 4 °C (1:1000 dilution). After three washes in TBS-T, membranes were incubated with HRP-conjugated anti-rabbit secondary antibodies (Cell Signaling, Danvers, MA, USA) for 2 h and washed in TBS-T. Signals were visualized after incubation with enhanced chemiluminescence kit (Bio-Rad, Hercules, CA, USA) by Chemidoc XRS+ (Bio-Rad, Hercules, CA, USA). Image analysis was performed using Image Lab™ 6.0 software (Bio-Rad, Hercules, CA, USA). Measurement of GAPDH content was used as loading control. Briefly, membranes were incubated with Restore Stripping Buffer for 15 min at room temperature (Thermo Scientific, Waltham, MA, USA), followed by incubation with anti-GAPDH primary antibody (1:5000 dilution, overnight at 4 °C), HRP-conjugated anti-rabbit secondary antibody (1:2000 dilution, 2 h at room temperature), and signal detection, as described previously.

### 2.8. SP-like Immunoreactivity

Human interventricular septum samples were homogenized in 1 mL of 20 mM phosphate buffer (K_2_HPO_4_ and KH_2_PO_4_, pH: 7.2) and 10 µL protease inhibitor (Gordox, 10,000 KIE/mL, Gedeon Richter Plc, Budapest, Hungary) with tissue homogenizer device (IKA T25 Digital ULTRA TURRAX). This was followed by centrifugation at 10,000 rpm at 4 °C for 15 min. The supernatant was collected and pooled at −80 °C.

Substance-P-like immunoreactivity was measured by the specific and sensitive radioimmunoassay method as described in detail in earlier publications [40] using the substance P competitive radioimmunoassay kit (cat.nr. RK-061-05, Phoenix Pharmaceuticals, Inc., Burlingame, CA, USA). Here, we only summarize the protocol briefly. Reconstituted positive controls and standards, 100 mL of tissue homogenates in duplicates, and 100 µL antiserum were incubated overnight at 4 °C in test tubes. Then, 100 µL 125I-labelled SP as tracer was added to the tubes on the consecutive day and additional overnight incubation was performed at 4 °C. Goat anti-rabbit IgG serum and normal rabbit serum were added to the designated tubes on the following day with a 90 min incubation period. Immunocomplexes were collected with centrifugation at 3000 rpm for at least 20 min at 4 °C, supernatant was carefully discarded, and pellet cpm was measured by g-counter (Gamma NZ-310, Budapest, Hungary). The results were expressed as fmol SP-like immunoreactivity per mg total protein weight.

### 2.9. NPY-like Immunoreactivity

Determination of NPY concentration in human interventricular septum samples was performed with RayBio^®^ Human/Mouse/Rat Neuropeptide Y competitive Enzyme Immunoassay Kit (cat.nr.: EIA-NPY, RayBiotech Life Inc., Peachtree Corners, GA, USA) according to the manufacturer’s instructions. We describe here the brief summary of the assay protocol. First, 100 µL of anti-NPY antibody solution was added to each well and 1.5 h of incubation with gentle shaking was performed at room temperature. After the incubation, the solution was removed and the wells were washed with 200–300 µL of 1× wash buffer solution, repeated four times. After the last washing step, the wash buffer was completely removed and the plate was inverted and blotted against clean paper towels. Next, 100 µL of standard reagents, positive control, and samples were added to the appropriate wells of the plate. The wells were covered and incubated at 4 °C overnight with gentle shaking. The solutions were removed, and the wells were washed four times with 1× wash buffer solution. To each well, 100 µL of prepared HRP-streptavidin solution was added and the plate was incubated for 45 min at room temperature with gentle shaking. After the next washing step, 100 µL of TMB One-Step Substrate Reagent was added to each well and incubation was performed for 30 min at room temperature, protecting the assay from light, with gentle shaking. At the end of the incubation period, 50 µL of stop solution was added to each well and color intensity measurement was performed at 450 nm with Labsystems DC plate reader immediately. Results were expressed as ng of NPY/mg of total protein content of the samples. The total protein concentration was measured with a bicinchoninic acid assay kit (Thermo Scientific Pierce Protein Research Products, Rockford, IL, USA) using bovine serum albumin as a standard.

### 2.10. Statistical Analysis

Statistical analyses were performed and graphs were created using GraphPad Prism version 8 (GraphPad Software, San Diego, CA, USA). One-way analysis of variance (ANOVA), two-way ANOVA, unpaired *t*-test, and Mann–Whitney test were used to find statistically significant differences. Differences were considered significant at values of *p* < 0.05. Unless noted otherwise, all data represent the mean ± SEM.

## 3. Results

A large-scale, multicenter, randomized, double-blind, and placebo-controlled phase 4 clinical trial called SAVOR-TIMI 53 aimed to assess the cardiovascular safety, efficacy, and potential cardioprotective benefits of saxagliptin, a DPP4 inhibitor [27]. According to the conclusions of that study, saxagliptin was found to be neutral in most of the primary and secondary end-points except that the heart-failure-associated hospitalization rate increased in the saxagliptin-treated patients compared to the control group. This result raised the question whether saxagliptin exerts any potentially harmful effect on the cardiovascular system.

### 3.1. Protein Expression of DPP4 and NPY Decreased in Human Failing Heart Samples

**Figure 1:**
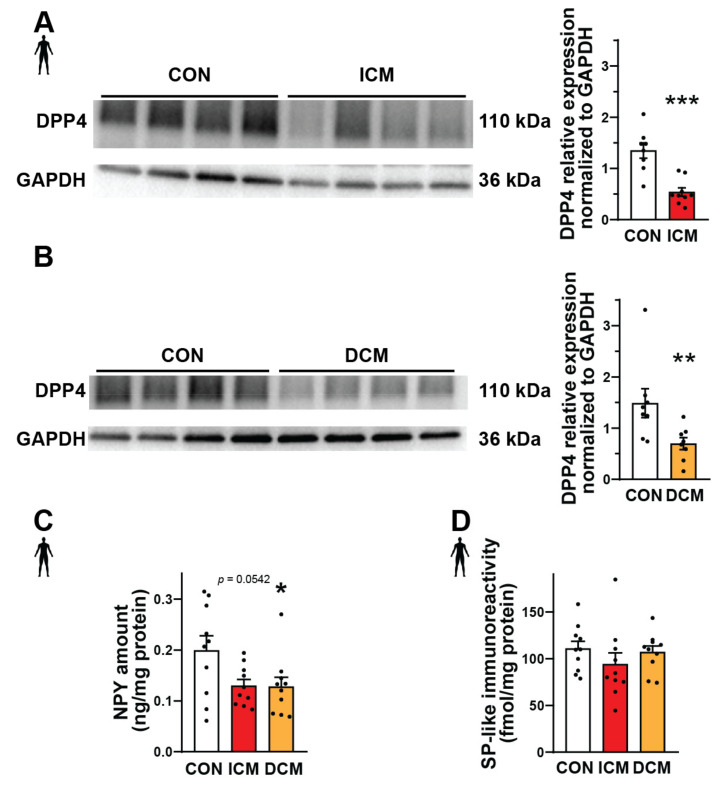
Protein expression of DPP4 and neuropeptides in failing human heart samples. Western blot analysis of DPP4 enzyme (**A**,**B**), ELISA (**C**), and radioimmunoassay (**D**). Quantification (**A**−**D**) of the DPP4 and neuropeptide substrates (NPY and SP) content in interventricular septum samples of healthy patients (CON) or patients with ischemic (ICM) or dilated cardiomyopathy (DCM). One-way ANOVA with Tukey’s post hoc test, unpaired *t*-test, and Mann–Whitney test * *p* < 0.05, ** *p* < 0.01, *** *p* < 0.001 vs. CON, group size: n = 8–10. Data are expressed as mean ± SEM.

Substance P and neuropeptide Y are important substrates of DPP4 that can mediate various detrimental effects in cardiovascular diseases [41,42,43]. Therefore, here we performed Western blot, ELISA, and radioimmunoassay experiments in order to measure the expression of DPP4, NPY, and SP at the protein level in interventricular septum samples from failing human hearts (patients with ischemic cardiomyopathy (ICM) or dilated cardiomyopathy (DCM)) and also from healthy control (CON) hearts (detailed patients’ characteristics in Supplementary Tables S1 and S2). We have found that the expression of DPP4 decreased significantly both in the ICM and DCM groups compared to the control (Figure 1A,B). NPY showed a significantly decreased expression in the DCM group and a decreasing tendency in the ICM group compared to the control (Figure 1C). The expression of SP remained around a similar level in each heart sample (Figure 1D).

After the successful measurements of the protein expressions, our following aim was to assess which cell types express the DPP4 enzyme.

### 3.2. DPP4 mRNA Is Primarily Localized in Cardiomyocytes of the Human Left Ventricle

**Figure 2:**
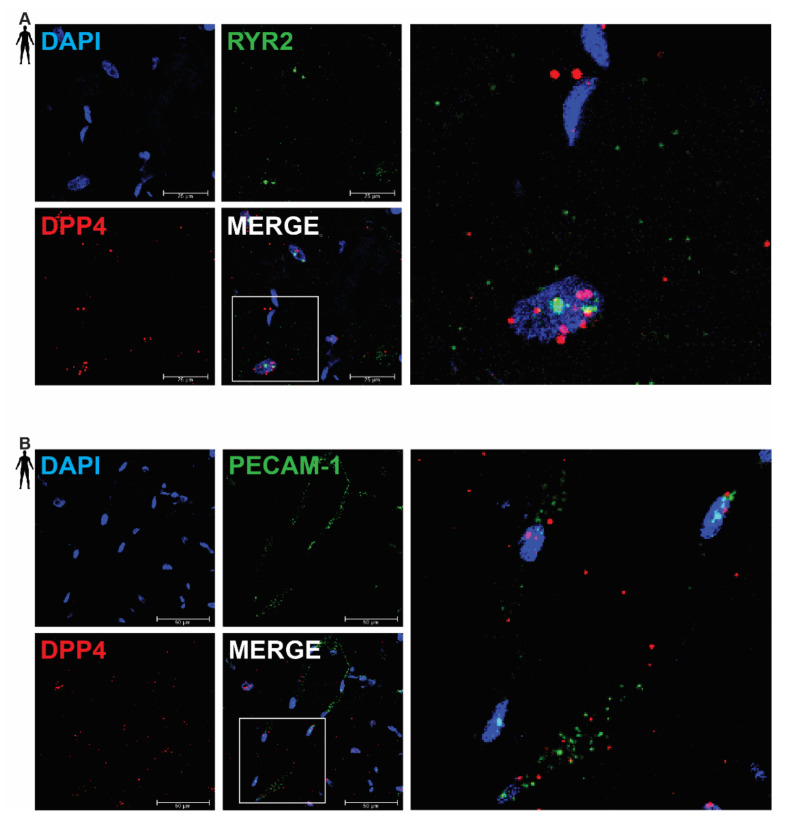
Representative confocal microscopy images of RNA Scope^®^-DPP4 mRNA expression in human control left ventricle. Nuclei were stained with DAPI (blue). Fluorescein-labeled tyramide (green) was used to visualize mRNA of RYR2 (cardiomyocyte marker, (**A**)) or PECAM-1 (endothelium marker, (**B**)) and Cy3-labeled tyramide (red) was used to visualize mRNA of DPP4, respectively. Scale bar represents 25 or 50 µm. (RYR2: ryanodine receptor 2, PECAM-1: platelet endothelial cell adhesion molecule-1).

**Figure 3:**
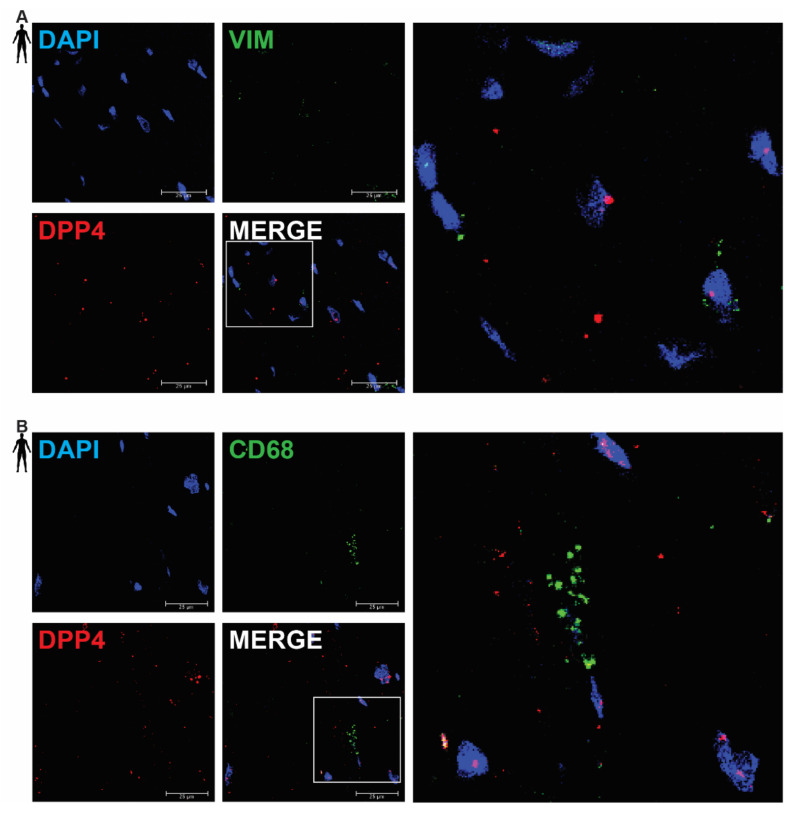
Representative confocal microscopy images of RNA Scope^®^-DPP4 mRNA expression in human control left ventricle. Nuclei were stained with DAPI (blue). Fluorescein-labeled tyramide (green) was used to visualize mRNA of VIM (fibroblast marker, (**A**)) or CD68 (macrophage marker, (**B**)) and Cy3-labeled tyramide (red) was used to visualize mRNA of DPP4, respectively. Scale bar represents 25 µm. (VIM: vimentin, CD68: cluster of differentiation 68).

In order to clarify the cell-type-specific expression of DPP4, RNA Scope^®^ in situ hybridization assay was performed on left ventricular tissue slides of healthy humans. Expression of the mRNA of DPP4 was shown primarily in RYR2 mRNA-positive cardiomyocytes (Figure 2A) and it was also detected to some extent in PECAM-1 mRNA-positive endothelial cells (Figure 2B), but not in VIM mRNA-positive fibroblasts (Figure 3A) and CD68 mRNA-positive macrophages (Figure 3B).

There was no detectable signal on the negative control slides (Supplementary Figure S1). These observations indicate that DPP4 is primarily expressed in cardiac myocytes and endothelial cells in the heart tissue.

### 3.3. DPP4 Inhibition and Neuropeptide Substrates Do Not Affect Cell Viability

**Figure 4:**
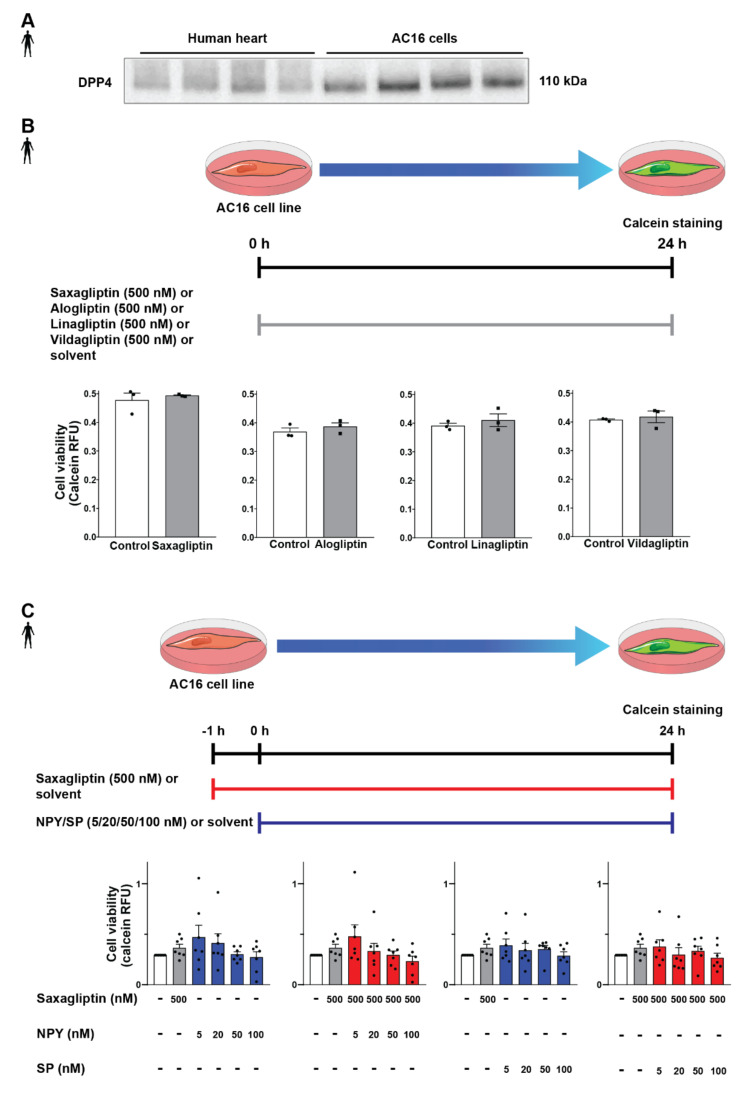
Effect of DPP4 inhibition and/or neuropeptide substrates on the viability of AC16 cells. Western blot analysis of DPP4 (**A**) in healthy human left ventricle samples and AC16 cells. In vitro treatment protocol with various gliptins on AC16 cell line and cell viability (calcein assay) results (**B**). In vitro treatment protocol with neuropeptides and their combined administration with saxagliptin and their effect on the viability of AC16 cells (**C**). One-way ANOVA, Tukey’s post hoc test, and unpaired *t*-test. Data are presented as mean ± SEM. Group sizes: (**B**) n = 3 from 1 independent experiment, (**C**) n = 7 from 7 independent experiments. RFU: relative fluorescence unit.

After successful determination of DPP4 and its substrates’ expression profile, we tested the potential detrimental effects of DPP4 inhibition and/or its substrates (SP, NPY) on cardiomyocytes. In order to begin the in vitro cell culture experiments, a proper cell culture model was required. We chose the AC16 cell line because these cells express the DPP4 enzyme at the protein level according to the results of the Western blot measurements (Figure 4A), also confirming our RNA Scope^®^ results. First, we hypothesized that direct cytotoxic effect of gliptins might be responsible for the slightly increased cardiovascular risk observed in SAVOR-TIMI, thus we performed the cell viability test on AC16 cells treated with saxagliptin or other clinically used gliptins. We have found that DPP4 inhibition by any of the tested gliptins (saxagliptin, vildagliptin, linagliptin, alogliptin) alone, at a concentration of 500 nM, does not have a toxic effect on cell viability of AC16 cells (Figure 4B). Next, we shifted our focus to investigating whether reduced DPP4 enzyme activity by gliptins increases the potential cytotoxic effects of its substrates NPY and SP. Thus, we treated the cells with NPY or SP or in combination with saxagliptin. We have found that neither neuropeptides (NPY or SP) alone nor in combination with saxagliptin can reduce the viability of AC16 cells (Figure 4C). These results indicate that saxagliptin and the neuropeptide substrates of DPP4 do not influence the viability of healthy cells; therefore, a different approach and model are required to test the potential cardiotoxic effect of saxagliptin.

### 3.4. Both Saxagliptin and the Neuropeptides Alter the Migration Capacity of Cardiac Fibroblasts

**Figure 5:**
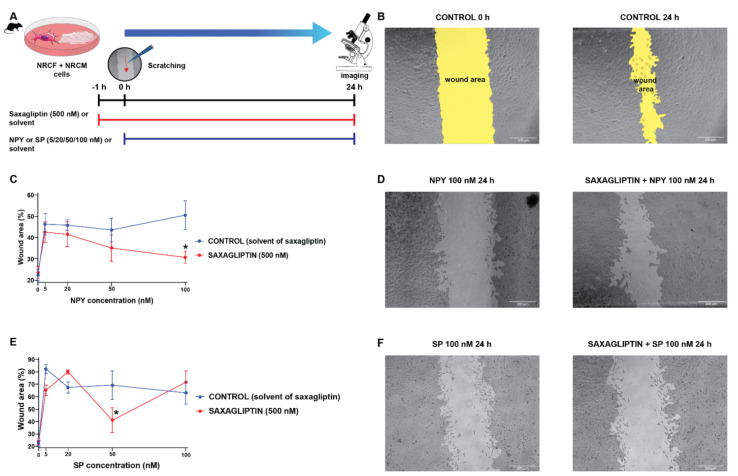
The cell migration speed in saxagliptin- and neuropeptide-treated cardiomyocyte/fibroblast co-culture model. Scratch assay treatment protocol (**A**) and the results of the treatment with NPY (**C**) and SP (**E**). Representative bright field microscope images of control (**B**), NPY-treated (**D**), and SP-treated groups (**F**). Two-way ANOVA, * *p* < 0.05 vs. CON. Group sizes: n = 8–22 from 3–5 independent experiments. Data are presented as mean ± SEM in percentage of the wound area compared to each corresponding 0 h (baseline) values.

Literature data suggest that SP and NPY may exert their harmful cardiovascular effects by the modulation of fibroblast activities [11,12,44,45]. Accordingly, we continued our experiments with a co-culture model of primary neonatal rat cardiomyocytes and cardiac fibroblasts, and performed scratch assay (which is widely used to model in vitro cell migration and wound healing) experiments (Figure 5A,B) with the same treatment protocol (neuropeptides and/or saxagliptin) as described above (Figure 5A,C).

We have found that administration of either NPY (Figure 5C,D) or SP (Figure 5E,F) reduces the migration speed of the cells significantly compared to the control groups (CONTROL and SAXAGLIPTIN). At the highest concentration of NPY (100 nM), the administration of saxagliptin (500 nM) restored the migration capacity of fibroblasts. In the case of the SP administration, there was no meaningful difference caused by the administration of saxagliptin.

## 4. Discussion

The surprising results of the SAVOR-TIMI 53 [27] clinical trial revealed that the heart-failure-associated hospitalization rate is increased in the saxagliptin-treated patients. These results point to the importance of cardiovascular safety testing in preclinical disease models to reveal the mechanisms of this clinically relevant cardiotoxicity. Therefore, in our present study, we focused on the investigation of the potential role of saxagliptin and the neuropeptide substrates of DPP4 in this phenomenon. Here, we showed that the protein expression of DPP4 and NPY decreased in the cardiac tissue of heart failure patients. Moreover, we also demonstrated by in situ hybridization that DPP4 mRNA is expressed mainly by cardiomyocytes. Interestingly, we have found that neither DPP4 inhibition alone nor in combination with neuropeptides such as NPY and SP affected the viability of AC16 cells, suggesting that the direct cytotoxic effect might not be primarily responsible for the observed adverse outcome in the clinical scenario [27]. In contrast, neuropeptides decreased cell migration speed in the co-culture of primary neonatal rat cardiomyocytes and cardiac fibroblasts. However, saxagliptin co-administration restored fibroblast migration speed in comparison to NPY. These results showed that DPP4 inhibition by saxagliptin and the increased level of neuropeptides in heart failure can modulate fibroblast migration; therefore, this may interfere with adaptive cardiac remodeling in heart failure, indicating a potential hidden drug cardiotoxicity mechanism [46].

Dipeptidyl-peptidase-4 is a pivotal enzyme in the degradation of various substances such as incretins, neuropeptides, and chemokines, among others. DPP4 inhibitors have become important drugs for the treatment of type 2 diabetes due to the improved regulation of glycemia through the increased glucagon-like peptide-1 levels. In addition, expression of DPP4 in the vascular endothelial cells raised its potential role in regulating vascular functions as well. Experimental results with various DPP4 inhibitors proved that DPP4 may play a role in the modulation of nitric oxide release [47] or reduce the severity of atherosclerotic lesions [48]. However, its role in regulating cardiac myocyte and fibroblast functions has not been investigated so far. We provide here the first evidence that the protein expression of DPP4 and NPY decreased in cardiac tissue samples of heart failure patients. This decrease in DPP4 expression was not related to the etiology of heart failure (ischemic or non-ischemic origin). Our findings are in line with previous data showing that cardiac tissue NPY protein expression is decreased in rats with volume-overload-induced heart failure, while in parallel, these studies revealed that the circulating level of NPY is increased due to heart failure [13]. Further supporting our results, Ajijola et al. have found that NPY immunoreactivity is decreased, but its mRNA expression did not change in the stellate ganglia of heart failure patients compared to healthy controls, suggesting increased release of NPY from the stellate ganglia [49].

We identified here that the DPP4 mRNA is localized mainly in cardiomyocytes and endothelial cells of the healthy human left ventricle. Previous studies revealed that DPP4 is widely expressed on the surface of various cell types, including leukocytes and epithelial or endothelial cell populations in several organs [50,51,52,53,54]. In line with these findings, we have also confirmed the presence of DPP4 mRNA in endothelial cells in the human heart as well. Our data highlight that cardiomyocytes could be a potential target cell type for saxagliptin, thus DPP4 inhibition in cardiomyocytes may play a role in mediating harmful effects.

The cardiovascular safety of saxagliptin in heart failure is quite controversial according to the currently available, conflicting literature data. Most of the clinical investigations and meta-analyses suggest that saxagliptin is harmful or at least shows controversial cardiovascular safety [55,56,57,58], while others found saxagliptin to be neutral [59,60,61], and there are several preclinical results on cardioprotective properties of DPP4 inhibitors [62,63,64]. Therefore, we investigated whether DPP4 inhibition in the presence of neuropeptide substrates could affect cardiomyocyte viability in vitro. We found that neither DPP4 inhibition alone nor in combination with neuropeptides exerts any cytotoxic effect on the human cardiomyocyte-like AC16 cells. According to the literature data, effects of saxagliptin treatment were investigated in various models of diabetes and/or acute cardiac damages. It was found that saxagliptin treatment exerted a direct cytoprotective effect in vitro against glucose and oxygen depletion and reoxygenation in cultured primary human brain microvascular endothelial cells [65]. Additionally, other groups demonstrated that saxagliptin was also able to decrease oxidative stress through regulation of endothelial nitric oxide synthase in a Goto-Kakizaki rat model of non-obese type 2 diabetes [66]. Saxagliptin also ameliorated cardiac diastolic dysfunction in isoproterenol-treated rats [63] and reduced cardiac ischemia-reperfusion injury in an ex vivo rat model of I/R injury with type 2 diabetes [64]. The discrepancy between the clinical and preclinical data is potentially due to differences in the mechanisms involved in the experimental models, since the majority of studies focus on diabetic models and acute cardiac damage, but not on chronic conditions such as heart failure.

Dipeptidyl-peptidase-4 inhibition by sitagliptin has been reported to decrease collagen deposition, and activation of pro-fibrotic signaling in rat hearts potentially improves fibrosis in heart failure [67]. These data suggest that the DPP4 enzyme and its substrates might be important in cardiac fibrosis, cell migration, and remodeling. Therefore, next, we investigated their potential effect on the migration speed of fibroblast cells and their potential interfering effect with adverse cardiac remodeling in a cardiomyocyte-fibroblast co-culture scratch assay model. We have found that administration of either NPY or SP significantly reduces the migration speed of the cells compared to the vehicle-treated controls. At the highest concentration of NPY, saxagliptin administration restored the migration capacity of fibroblasts. Interestingly, in other cell types, NPY has been shown to stimulate migration of human umbilical endothelial cells [68], to promote ischemic angiogenesis and vascularization in the rat ischemic hind limb [69], and to increase motility of neuroblastoma cells [70]. Our finding suggests that NPY might act differently on cardiac fibroblasts, reducing their migration and likely preventing cardiac fibrosis. Interestingly, saxagliptin reverses the effect of NPY on fibroblast migration; therefore, saxagliptin in the presence of NPY may exert harmful profibrotic effects.

In conclusion, we have shown that the expression of DPP4 enzyme and the neuropeptide tone is altered in human failing hearts. Although DPP4 inhibition neither alone nor in combination with neuropeptides has any detectable cytotoxic effect in vitro, neuropeptides inhibited cell migration in primary neonatal rat cardiomyocyte/fibroblast co-cultures, which is reversed by saxagliptin co-administration. Our results highlight that saxagliptin and NPY may interfere with cardiac tissue remodeling and thus play a role in the pathophysiological mechanisms of end-stage chronic heart failure. We believe that the DPP4 enzyme could exert a compensatory function against the altered neuropeptide tone caused by the elevated sympathetic activity in heart failure. Inhibition of DPP4 by saxagliptin could impair this adaptive mechanism, and thereby exacerbate myocardial damage, although further experiments are required to more deeply understand the potential role of other DPP4 substrates and the possible direct toxic effects of saxagliptin.

## Data Availability

The datasets used and/or analyzed during the current study are available from the corresponding author on reasonable request.

## Supplementary Materials

The following supporting information can be downloaded at: https://www.mdpi.com/article/10.3390/biomedicines10071573/s1, Table S1: Patient characteristics in Western blot experiments; Table S2: Patient characteristics in radioimmunoassay and ELISA experiments; Figure S1: Representative confocal microscopy images of RNA Scope^®^ negative control in histological samples of human control left ventricle.

## Abbreviations

**ANOVA**	analysis of variance
**CD68**	cluster of differentiation 68
**CON**	control
**Cy3**	cyanine 3
**DAPI**	4′,6-diamidino-2-phenylindole
**DCM**	dilated cardiomyopathy
**DMEM**	Dulbecco’s modified eagle medium
**DMSO**	dimethyl sulfoxide
**D-PBS**	Dulbecco’s phosphate-buffered saline
**DPP4**	dipeptidyl-peptidase-4
**ELISA**	enzyme-linked immunosorbent assay
**FBS**	fetal bovine serum
**GAPDH**	glyceraldehyde 3-phosphate dehydrogenase
**HBSS**	Hank’s balanced salt solution
**HRP**	horseradish peroxidase
**ICM**	ischemic cardiomyopathy
**mRNA**	messenger RNA
**PECAM-1**	platelet endothelial cell adhesion molecule 1
**RFU**	relative fluorescent unit
**RYR2**	ryanodine receptor 2
**SEM**	standard error of the mean
**VIM**	vimentin
