# Supplementary material for: Saxagliptin Cardiotoxicity in Chronic Heart Failure: The Role of DPP4 in the Regulation of Neuropeptide Tone
Source: Biomedicines. 2022 Jul 1;10(7):1573. doi: 10.3390/biomedicines10071573 (PMC9312997; doi:10.3390/biomedicines10071573)
Supplement: Supplementary file 1 [file biomedicines-10-01573-s001.zip › biomedicines-1744334-supplementary.pdf]

# Supplementary Material

# Saxagliptin cardiotoxicity in chronic heart failure: the role of DPP4 in the regulation of neuropeptide tone

**Imre Vörös[1,2,3], Zsófia Onódi[1,2,3], Viktória Éva Tóth[1,2,3], Tamás G. Gergely[1,2,3], Éva Sághy[1], Anikó Görbe[1,4], Ágnes Kemény[5,6,7], Przemyslaw Leszek[8], Zsuzsanna Helyes[5,6,9], Péter Ferdinandy[1,4], Zoltán V. Varga[1,2,3*]**

[1]Cardiometabolic Research Group and MTA-SE System Pharmacology Research Group, Department of Pharmacology and Pharmacotherapy, Semmelweis University, 1085 Budapest, Hungary; voros.imre@med.semmelweis-univ.hu (I.V.); onodi.zsofia@med.semmelweis-univ.hu (Z.O.); toth.viktoria@med.semmelweis-univ.hu (V.É.T.); gergely.tamas@med.semmelweis-univ.hu (T.G.G.); saghy.eva@med.semmelweis-univ.hu (É.S.); gorbe.aniko@med.semmelweis-univ.hu (A.G.); peter.ferdinandy@pharmahungary.com (P.F.); varga.zoltan@med.semmelweis-univ.hu (Z.V.V.);

[2]HCEMM-SU Cardiometabolic Immunology Research Group, Semmelweis University, 1085 Budapest, Hungary;

[3]MTA-SE Momentum Cardio-Oncology and Cardioimmunology Research Group, Semmelweis University, 1085 Budapest, Hungary;

[4]Pharmahungary Group, 6722 Szeged, Hungary;

[5]Szentágothai János Research Centre, University of Pécs, 7624 Pécs, Hungary; kemeny.agnes@pte.hu (Á.K.); zsuzsanna.helyes@aok.pte.hu (Z.H.);

[6]Department of Pharmacology and Pharmacotherapy, Medical School, University of Pécs, 7624 Pécs, Hungary;

[7]Department of Medical Biology, University of Pécs, 7624 Pécs, Hungary;

[8]Department of Heart Failure and Transplantology, Cardinal Stefan Wyszyński National Institute of Cardiology, 04-628 Warszawa, Poland; przemyslaw.leszek@ikard.pl (P.L.);

[9]PharmInVivo Ltd. 7629 Pécs, Hungary

* Correspondence: varga.zoltan@med.semmelweis-univ.hu; Tel.: +36-1-2104416

## SUPPLEMENTARY TABLES

### Supplementary Table S1 – Patient characteristics in Western blot experiments

| Parameter | CON | ICM | DCM |
|---|---|---|---|
| **Gender** (male/female) | 6/2 | 8/1 | 8/0 |
| **Age** (year) | 33 (23-52) | 58 (39-66) | 48 (27-58) |
| **BMI** (kg/m$^2$) | 25 (20-32) | 27 (20-33) | 29 (21-40) |
| **Ejection fraction** (%) | - | 21 (13-33) | 19 (5-33) |
| **N-terminal pro-BNP** (pg/ml) | - | 3328 (338-8024) | 5308 (1001-14750) |
| **Medications** | | | |
| **Norepinephrine** | 7 | 0 | 1 |
| **ACE inhibitors** | 0 | 7 | 6 |
| **Beta receptor blockers** | 1 | 9 | 8 |
| **Mineralocorticoid receptor inhibitors** | 0 | 9 | 2 |
| **Furosemide** | 1 | 0 | 0 |
| **Mannitol** | 1 | 0 | 0 |
| **Digoxin** | 0 | 1 | 1 |
| **Mexiletine** | 0 | 1 | 1 |
| **Amiodarone** | 0 | 3 | 5 |
| **Anticoagulants** | 1 | 5 | 4 |
| **Antiplatelets** | 0 | 7 | 0 |
| **Dopamine** | 0 | 3 | 3 |
| **Dobutamine** | 0 | 3 | 4 |
| **Sildenafil** | 0 | 3 | 2 |
| **Statins** | 0 | 9 | 2 |
| **Allopurinol** | 0 | 1 | 3 |

Clinical parameters of the human interventricular tissue samples used in Western blot experiments. Data are expressed as mean and range. Abbreviations: CON: control; ICM: ischemic cardiomyopathy; DCM: dilated cardiomyopathy

**Supplementary Table S2 – Patient characteristics in radioimmunoassay and ELISA experiments**

| Parameter | CON | ICM | DCM |
|---|---|---|---|
| **Gender** (male/female) | 4/6 | 9/1 | 10/0 |
| **Age** (year) | 38 (17-52) | 62 (45-74) | 48 (27-60) |
| **BMI** (kg/m$^2$) | 26 (20-34) | 26 (20-33) | 27 (20-39) |
| **Ejection fraction** (%) | - | 21 (10-33) | 17 (5-25) |
| **N-terminal pro-BNP** (pg/ml) | - | 3071 (338-7699) | 8632 (1001 - 35000) |
| **Medications:** | | | |
| **Norepinephrine** | 10 | 0 | 1 |
| **ACE inhibitors** | 0 | 8 | 8 |
| **Beta receptor blockers** | 1 | 10 | 10 |
| **Mineralocorticoid receptor inhibitors** | 0 | 10 | 9 |
| **Furosemide** | 2 | 0 | 0 |
| **Mannitol** | 2 | 0 | 0 |
| **Digoxin** | 0 | 2 | 2 |
| **Mexiletine** | 0 | 1 | 1 |
| **Amiodarone** | 0 | 2 | 6 |
| **Anticoagulants** | 1 | 6 | 6 |
| **Antiplatelets** | 0 | 7 | 0 |
| **Dopamine** | 4 | 2 | 3 |
| **Dobutamine** | 0 | 2 | 4 |
| **Sildenafil** | 0 | 3 | 2 |
| **Statins** | 0 | 10 | 1 |
| **Allopurinol** | 0 | 4 | 3 |

Clinical parameters of the human interventricular tissue samples used in radioimmunoassay and ELISA experiments. Data are expressed as mean and range. Abbreviations: CON: control; ICM: ischemic cardiomyopathy; DCM: dilated cardiomyopathy

## SUPPLEMENTARY FIGURES

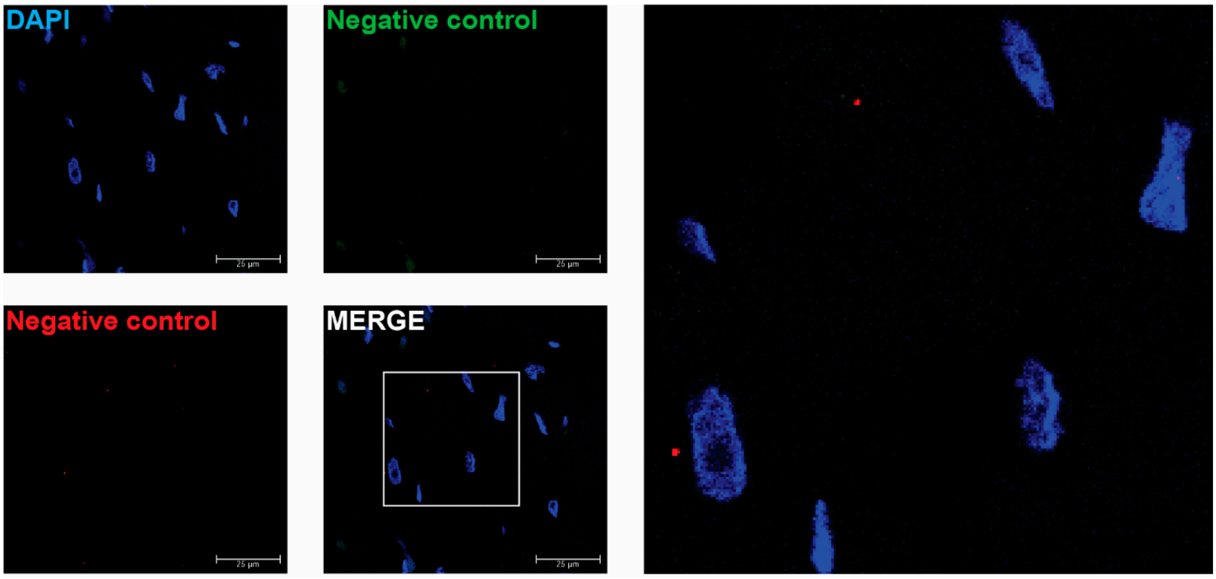


**Supplementary Figure S1 -** Representative confocal microscopy images of RNA Scope® negative control (green, red) in histological samples of human control left ventricle. Cells were counterstained with DAPI (blue). Scale bar: 25 µm.
